# Population Screening for Cancer in High-Income Settings: Lessons for Low- and Middle-Income Economies

**DOI:** 10.1200/JGO.18.00235

**Published:** 2019-02-04

**Authors:** Philippe Autier, Richard Sullivan

**Affiliations:** ^1^ University of Strathclyde Institute of Global Public Health at International Prevention Research Institute, Ecully, Lyon, France; ^2^ International Prevention Research Institute, Lyon, France; ^3^ King’s College London, London, United Kingdom

## Introduction

The primary goal of screening for cancer is to decrease deaths caused by cancer. National cancer screening in high-income countries (HICs) started in the 1960s, when nationwide Papanicolaou test screening was introduced in some countries (eg, Finland, Sweden) for cervical cancer screening. Since then, numerous screening methods have been proposed, even for rarer cancers like the testis cancer or neuroblastoma.

Many low- and medium-income countries (LMICs) have also started to introduce screening technologies and contemplate the adoption of nationwide screening policies. However, decision makers of LMICs should not overlook the accumulated experience points from high-income settings, such as discrepancies observed between efficacy of screening as shown by randomized trials conducted in ideal conditions and effectiveness of screening in populations as shown by population data; overdiagnosis, which is the detection of cancerous lesions that would not be clinical during the patient’s lifetime and the increased detection of cancerous lesions that were uncommon before screening (eg, the in situ or borderline cancers); overtreatment, which is the treatment of overdiagnosed screen-detected cancers or of screen-detected benign lesions of unknown clinical behavior; and the extra costs and loss in quality of life induced by false-positive screening results, overdiagnosis, and overtreatment. The ultimate consequence of these issues is that the appraisal of the benefit-to-harm balance of screening policies has proven to be much more difficult than anticipated in HICs, where significant structural, political, and economic advantages exist compared with resource-constrained settings, and a closer look at these major issues is particularly relevant for LMICs. In this article, we review the consistency between efficacy and effectiveness of screening for four cancers (cervical, colorectal, and breast cancers and the neuroblastoma) for which considerable experience has been gathered in HICs and what lessons there are in these experiences for LMICs.

## The logic of cancer screening

A critical appraisal of any cancer screening policy in any resource setting needs to bear in mind the basic mechanisms by which screening can reduce cancer mortality, which is the detection of cancer precursor lesions or of cancers at an earlier curable stage in asymptomatic individuals, before metastases have spread in lymph nodes or in distant organs. It follows that if a screening method is truly effective, then in populations where screening is widespread, decreases in incidence rates of advanced-stage cancers after screening introduction should be the first sign that screening contributes to declines in cancer mortality.^1^ This indicator has the advantage of being independent of the influence of therapies on cancer mortality. Likewise, in areas where patient management and access to effective therapies are similar, the cancer-specific mortality rates should decline more rapidly in areas with high participation in screening than in areas with less screening.^2^

## Screening for cervical and colorectal cancer

All randomized trials on screening for cervical and colorectal cancer have demonstrated that commonly recommended screening methods are able to decrease the incidence of advanced-stage cancers and the mortality associated with these cancers (Table 1).^3^ The randomized trials on cervical cancer screening have all been conducted in India and have documented the efficacy of visual inspection, cytology screening, and human papillomavirus screening.^4-7^ The conclusions of these trials are highly relevant to LMICs, where nine out of 10 (87%) cervical cancer deaths occur.^8^

**TABLE 1:**
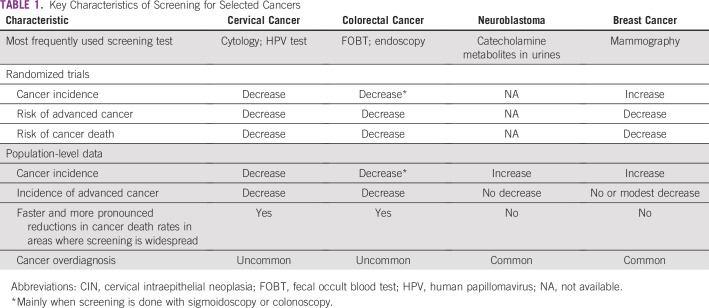
Key Characteristics of Screening for Selected Cancers

The epidemiologic studies have consistently documented that screening of populations for colorectal and cervical cancers contributes to decreasing the incidence of advanced-stage cancers and the mortality associated with these two cancers (Table 1).^9-11^ The overdiagnosis is deemed to be limited. Hence, for these two cancers, results of randomized trials and of population studies are in good agreement for both the incidence of advanced-stage cancers and for cancer-specific mortality.^12-15^

## Screening for neuroblastoma

Neuroblastoma is the most common extracranial solid tumor in childhood in HICs, where it accounts for 10% of pediatric cancers.^16^ Although the disease is also clearly present in LMICs, its quantification remains difficult because of many undiagnosed cases. However, considering the young demographic profiles of LMICs, the overall number of neuroblastomas is certainly much greater in these countries than in HICs. Whether that child is born in an HIC or an LMIC, neuroblastoma is often metastatic at diagnosis and fatality is high, mainly when the diagnosis is made after the first year of life. Children with neuroblastoma have increased concentrations of catecholamine metabolites in the urine. It was therefore proposed to screen 6-month- to 1-year-old children for the detection of high concentration of urine catecholamine metabolites. Screening programs were in place from 1985 to 2004 in Japan, from 1995 to 2000 in six of 16 states in Germany, and from 1989 to 1994 in Quebec (Canada).^17^ It was soon evidenced that in areas with screening, the incidence of advanced neuroblastoma remained at the level prevailing before screening introduction, and neuroblastoma mortality did not decrease more rapidly than in areas where screening was uncommon.^18-20^ At the same time, three- to four-fold increases in the incidence of small-size neuroblastoma were observed,^20,21^ which revealed the reality of overdiagnosis (ie, the detection of nonprogressing or self-regressing occult neuroblastoma that would have not been life-threatening during the individual’s lifetime had the urine testing not been carried out).^21^ As a consequence of the unfavorable benefit-to-harm balance (Table 1), neuroblastoma screening programs were discontinued after 2000 in all countries where this screening had been implemented.^22^

## Screening mammography for breast cancer

Screening mammography has been implemented in most HICs since the 1990s. Recommendations for screening mammography were backed by five randomized trials conducted in Sweden (four trials) and in England (one trial). Two trials in Canada found no reduction in breast cancer mortality associated with mammography screening.^23^ All breast screening trials being considered, a 20% reduction in the risk of breast cancer in women 40 to 75 years of age could be expected after 13 years of follow-up.^23^ Health professionals involved in randomized trials on mammography screening have proposed to monitor the incidence of advanced breast cancer for evaluating the effectiveness of this screening.^2,24-26^ There was thus great expectation in 1990 to 2005 that screening mammography would change the burden of breast cancer in a way that resembles changes observed for colorectal and cervical cancer, except for the incidence of localized cancers, because of the propensity of mammography screening to detect occult in situ and invasive cancers that would not be clinical during women’s lifetime (ie, overdiagnosis).

But, contrary to expectations, in areas where mammography screening has been in place for 20 to 35 years (eg, the United States, the Netherlands, and Copenhagen in Denmark), and where 70% to 80% of women have attended largely more screening rounds than the two to four rounds offered by most screening mammography trials, the incidence of advanced-stage breast cancers has remained fairly stable over time, including that of cancers with metastatic spread in distant organs at diagnosis.^27-29^ Hence, the screen detection of occult in situ or invasive localized cancers during a substantial number of years did not lead to (or led to only fewer) less-advanced–stage cancers in subsequent years. As a logical upshot, in areas where access to therapies is the same, breast cancer mortality reductions have never been quicker and more pronounced in areas with early than in areas with late implementation of screening.^30^ These epidemiologic features are independent of age at screening start and of the time between screening rounds. According to randomized trials, 20% to 30% of screen-detected in situ and invasive cancers could be considered as overdiagnosis.^31^ Thus, the balance between effectiveness and overdiagnosis of screening mammography in populations resembles the balance of neuroblastoma screening and not that of screening for cervical and colorectal cancer (Table 1). This is a critical issue for LMICs considering adopting population mammographic screening, where many are under considerable political and emotional pressure to allocate limited resources to what is a highly expensive program.

## Reasons for the discrepancy between breast screening efficacy and effectiveness

Why are the results of randomized trials, especially of Swedish trials, hardly evidenced in populations? One answer is to consider that methods based on the monitoring and comparison of trends of advanced-stage cancer and cancer-specific mortality would not inform on the true effectiveness of screening mammography, and one should give the preference to observational studies.^32^ However, why would methods that provided evidence for the effectiveness of screening for cervical and colorectal cancer, and for the ineffectiveness of screening for neuroblastoma, suddenly no longer be valid for evaluating the effectiveness of screening mammography? Moreover, observational studies for the evaluation of screening effectiveness are highly susceptible to biases, which led the International Agency for Research on Cancer Handbook of 2002 on breast cancer screening to issue a warning: “observational studies based on individual screening history, no matter how well designed and conducted, should not be regarded as providing evidence for an effect of screening.”^2^(p91)

The alternative answer is to admit that the methods commonly recommended for evaluating cancer screening effectiveness^1,2,24^ show the limited effectiveness of screening mammography. If so, then the credibility of randomized trials that found a reduced risk of breast cancer death associated with breast screening is to be questioned. With few exceptions,^23^ systematic reviews have taken the results of breast screening trials at face value, considering that the methodological imperfections were not likely to invalidate their results.^2,31^ However, careful comparisons of randomized trials on cancer screening have documented that the design and the statistical analysis of all breast screening trials that suggested health benefit associated with screening were distinct from designs and statistical analyses used in trials on screening for other cancers.^30^ These methodological differences most probably led to overestimating the capacity of breast screening to reduce the risk of advanced-stage cancer and of breast cancer death.^3,30,33^ This is crucial, because simply repeating mammographic screening clinical trials in LMICs is not the answer, and indeed would undoubtedly be a significant waste of research funding. What is clear is that in limited-resource settings, addressing the social, political, and economic determinants of late presentation of patients with cancer is what matters, not mammographic screening, as well as putting in place high-quality, affordable breast cancer care.

## Discussion

This review documents the evidence that for cervix and colorectal cancers, the results from randomized trials have been evidenced in populations, with screening being able to reduce the incidence rates of advanced-stage cancers, followed by reductions in cancer-specific mortality. Screening can be contemplated in areas of LMICs where the burden of these two cancers is important and where resources allow sustainable good-quality screening. Such prospects need to be linked with context-specific enhancements of capacity and capability for managing both benign and malignant disease. Models and strategic thinking have already been forthcoming in contexts such as Zambia.^15^

The similarities in the limited effectiveness and overdiagnosis of screening mammography and screening for neuroblastoma raise legitimate concerns about recommending the adoption of screening mammography policies. One could argue that the comparison of screening for a common and for a rare cancer is probably not appropriate. However, small children and pregnant women are often systematically screened for more than 100 conditions that are even rarer than neuroblastoma.^34^ Moreover, the theory underlying screening of asymptomatic individuals is the same for any disease, irrespective of age, sex, location, and incidence.^35^ Last, the poor benefit-to-harm balance of screening for prostate cancer using the serum prostate specific antigen test has also been compared with that of screening for neuroblastoma.^36^

In this regard, LMICs should think of other health priorities (eg, cervix cancer screening and access to adequate curative services for patients with cancer) before mammography screening.^37^ Breast physical examination and breast self-examination could represent less-demanding screening methods, but their efficacy is still largely unknown. In one randomized trial on breast physical examination in India, the incidence rate of advanced-stage cancers remained unaltered in the 3 years after screening started.^38^ Hence, a research priority is to find new breast screening methods that can truly reduce the burden of advanced-stage cancers while causing minimal overdiagnosis.

## Data Availability

The following represents disclosure information provided by authors of this manuscript. All relationships are considered compensated. Relationships are self-held unless noted. I = Immediate Family Member, Inst = My Institution. Relationships may not relate to the subject matter of this manuscript. For more information about ASCO's conflict of interest policy, please refer to www.asco.org/rwc or ascopubs.org/jco/site/ifc. **Research Funding:** Sanofi (Inst) **Honoraria:** Pfizer **Consulting or Advisory Role:** Pfizer (Inst)
